# Negative first impression judgements of autistic children by non-autistic adults

**DOI:** 10.3389/fpsyt.2023.1241584

**Published:** 2023-10-06

**Authors:** Troy Q. Boucher, Julia N. Lukacs, Nichole E. Scheerer, Grace Iarocci

**Affiliations:** ^1^Department of Psychology, Simon Fraser University, Burnaby, BC, Canada; ^2^Department of Psychology, Wilfrid Laurier University, Waterloo, ON, Canada

**Keywords:** autism spectrum disorder, first impressions, autism bias, autism stigma, social competence

## Abstract

**Introduction:**

Although autism inclusion and acceptance has increased in recent years, autistic people continue to face stigmatization, exclusion, and victimization. Based on brief 10-second videos, non-autistic adults rate autistic adults less favourably than they rate non-autistic adults in terms of traits and behavioural intentions. In the current study, we extended this paradigm to investigate the first impressions of autistic and non-autistic children by non-autistic adult raters and examined the relationship between the rater's own characteristics and bias against autistic children.

**Method:**

Segments of video recorded interviews from 15 autistic and 15 non-autistic children were shown to 346 undergraduate students in audio with video, audio only, video only, transcript, or still image conditions. Participants rated each child on a series of traits and behavioural intentions toward the child, and then completed a series of questionnaires measuring their own social competence, autistic traits, quantity and quality of past experiences with autistic people, and explicit autism stigma.

**Results:**

Overall, autistic children were rated more negatively than non-autistic children, particularly in conditions containing audio. Raters with higher social competence and explicit autism stigma rated autistic children more negatively, whereas raters with more autistic traits and more positive past experiences with autistic people rated autistic children more positively.

**Discussion:**

These rapid negative judgments may contribute to the social exclusion experienced by autistic children. The findings indicate that certain personal characteristics may be related to more stigmatised views of autism and decreased willingness to interact with the autistic person. The implications of the findings are discussed in relation to the social inclusion and well-being of autistic people.

## 1. Introduction

Although many autistic children desire to make friends (1), they encounter difficulties when it comes to social isolation, exclusion, and victimisation more frequently than their non-autistic peers and children with other disabilities (2–5). The difficulties that autistic people face have been partly attributed to the stigma around autism. Stigma refers to a negative indicator or attribution attached to a particular characteristic or difference. This stigma may combine with negative attitudes towards this difference, contributing to the devaluation or discrimination against a person or a group (6). The stigma and negative perceptions towards autistic children may contribute to their exclusion by family members, teachers and peers in school, and people in their community. Autism stigma may lead someone to judge an autistic child and treat them differently. It is considered a stigma, even when these actions are not conscious and overt. For example, negative perceptions may contribute to non-autistic people misunderstanding autistic children's words or actions (7), telling autistic children to try to “fit in” (8), and, in more extreme cases, victimisation and violence (9). Repeated experiences of stigmatisation have a cumulative effect on the autistic person's wellbeing and contribute to the significantly higher rates of anxiety, depression, and suicidality in autistic people (10, 11).

First impressions are the rapid judgement of personality traits and social characteristics that are made after a brief initial exposure to a person or stimulus (12). Previous research studies indicate that non-autistic people form quick and strong negative first impressions of autistic people based on short de-contextualised videos (13–19). Participants in these studies watched 10-s videos, referred to as “thin-slices”, created by Sasson et al. (14) of autistic and non-autistic adults. Participants then completed a questionnaire to record their first impressions of the person in the video, rating them on their traits (i.e., attractiveness, awkwardness, intelligence, likeability, trustworthiness, and dominance) and behavioural intentions towards them (i.e., willingness to live near, hang out with, comfort sitting next to, and the likelihood of starting a conversation with the person in the video). Across these studies, autistic adults were consistently rated by non-autistic adults as being more awkward and less likeable than their non-autistic peers, even though the non-autistic raters were not informed of the diagnostic status of the individuals in the videos (14–18). Similarly, non-autistic adults have reported a disinclination to interact with the autistic people in these videos. These negative first impressions, if they are indicative of the implicit negative attitudes held about autism, may contribute to the social exclusion experienced by autistic people.

It is likely that differences in the verbal and non-verbal behaviours of autistic adults are identified as peculiar by non-autistic raters, leading to less favourable ratings of autistic people. In a study by Sasson et al. (14), non-autistic adults formed less favourable first impressions of autistic adults compared to non-autistic adults when exposed to a sample of their social communication in audio and/or visual formats. However, when presented with a transcript of the same audio or visual recordings, there were no differences in their first impressions between autistic and non-autistic adults. Similar negative first impression formation was found in the ratings of children by non-autistic adults. Grossman (13) used recordings of autistic and non-autistic children across different audio and video formats, including audio only, video only, audio with video, and still images. After each recording of a child was presented, participants responded with “yes” or “no” to indicate their perception of whether that child was socially awkward. Participants rated autistic children as socially awkward more often than non-autistic children across all audio-visual formats.

Furthermore, these negative first impressions of autistic adults appear to be related to the characteristics of the non-autistic rater. Raters, who scored higher on a questionnaire assessing explicit stigma, rated autistic people in short video clips less favourably on characteristics such as awkwardness, attractiveness, trustworthiness, dominance, likability, and intelligence (16). In a similar study with high-school-aged non-autistic raters (ages 15–19 years old), a higher self-rating of social competence was associated with greater negative perceptions of autistic adults (15). Other characteristics, such as quality and quantity of previous contact with autistic people, were also related to raters' attitudes towards autistic people (20).

Although previous research studies have already documented negative judgements of autistic children by non-autistic adults (13), this study aims to explore the factors that contribute to these negative judgements by varying the audio-visual formats through which the autistic child is perceived and by assessing the characteristics of the non-autistic examiner. In light of negative perceptions of autistic adults and children by non-autistic perceivers increasingly being reported in recent literature, the current study examined the first impressions of autistic and non-autistic children by non-autistic adults using a “thin slice” paradigm [see Grossman (13) and Sasson et al. (14)]. This research extends previous rese by examining the potential effect of audio-visual mediums (i.e., the effect of auditory, visual, and content cues within a de-contextualised conversational segment) on the formation of first impressions of autistic and non-autistic children. The study also extends previous research by examining the potential effect of the non-autistic rater's personal characteristics (i.e., explicit stigma, social competence, autistic traits, and past experiences with autistic people) on the formation of first impressions of autistic children.

The following hypotheses were proposed: first, non-autistic adult raters would rate autistic children less favourably than non-autistic children when evaluating personal characteristics and behavioural intentions across different audio-visual formats. Second, the following relationships would be supported: higher levels of social competence and explicit stigma would predict more negative first impressions of autistic children, while higher levels of autistic traits and quality and quantity of past experiences with autistic people would predict more positive first impressions of autistic children. We also explored differences in first impression ratings across audio-visual mediums given the mixed results reported by other researchers [i.e., Grossman (13) and Sasson et al. (14)] regarding the effect of these mediums.

## 2. Methods

### 2.1. Participants

We extracted stimuli from a set of 52 semi-structured interviews with autistic and non-autistic children (ages 6–11 years old) discussing their interests (21). Before the interview, parents provided consent for their child's audio and video recordings, the use of these recordings for future studies, and for use in publication, while the children provided assent for the interview. For the current study, the inclusion of each stimulus was determined by calculating the longest utterances of each stimulus participant. This was defined as the child speaking to the interviewer for at least 8 s without disclosing any personally identifying information (e.g., their name), without pausing for more than 3 s, and without being interrupted by the interviewer. In addition to these criteria, the stimuli were excluded if the stimulus participant had a diagnosis of intellectual disability, stood up or was seated out of frame during their longest utterance, or wore clothing that obscured their face. After eligibility screening, a total of 30 interviews were edited and used in the current study.

Of the 22 excluded stimulus participants, 17 (12 autistic and five non-autistic) were excluded because they did not meet the utterance inclusion criteria. One autistic stimulus participant was excluded because they had a diagnosis of intellectual disability. Two (one autistic and one non-autistic) stimulus participants were excluded because they were not sitting within the frame of the camera during the interview. One non-autistic stimulus participant was excluded because they wore a mask for the duration of the interview. One autistic stimulus participant was excluded because the camera malfunctioned during the interview and, thus, no video was recorded.

Fifteen autistic (mean age = 8.84, SD = 1.68) and 15 non-autistic (mean age = 8.77, SD = 2.11) children served as stimulus participants. These stimulus participants did not significantly differ in age or IQ between groups and had a similar makeup of gender and cultural background (see Table 1). Caregivers of the autistic children provided a copy of their child's diagnostic report to confirm a diagnosis of autism spectrum disorder (ASD). To receive a diagnosis of ASD in the Canadian province of British Columbia, one must complete the Autism Diagnostic Interview-Revised (ADI-R) and the Autism Diagnostic Observation Schedule (ADOS-2). Additionally, they must meet the criteria outlined in the Diagnostic and Statistical Manual of Mental Disorders, Fifth Edition (22). All autistic children met these criteria.

**Table 1:** Descriptive statistics and demographic information of stimulus participants.

	**Autistic (*n* = 15)**	**Non-autistic (*n* = 15)**			
**Variable**	**Mean (SD)**	**Mean (SD)**	***df***	***t***	***p***
Age	8.84 (1.68)	8.77 (2.11)	28	−0.097	0.923
WASI-II FSIQ-2	101 (16.57)	109 (11.83)	28	1.687	0.103
AQ Total Score	30.20 (6.24)	17.53 (8.20)	28	−4.758	<0.001
SRS-2 Total T Score	70.87 (11.24)	49.67 (10.73)	28	−5.283	<0.001
	***n*** **(%)**	***n*** **(%)**			
**Gender**					
Men	9 (60%)	8 (53%)			
Women	6 (40%)	7 (47%)			
**Ethnicity**					
East Asian	6 (40%)	5 (33%)			
Latin American	1 (7%)	2 (13%)			
White/European	8 (53%)	8 (53%)			

n = 30.

WASI-II FSIQ-2, Wechsler Abbreviated Scale of Intelligence, 2nd Edition, Two Scale Intelligence Quotient; AQ, Autism-Spectrum Quotient; SRS-2, Social Responsiveness Survey, 2nd Edition.

Rating participants were recruited from introductory university psychology classes and were compensated with course credit. A total of 346 undergraduate students (ages 17– 49 years, mean age = 19.44, SD = 2.84) participated in the current study as raters. Participants were randomly assigned to one of five categories: audio with video (*n* = 93, mean age = 19.32, SD = 1.69), audio only (*n* = 62, mean age = 20.98, SD = 4.98), video only (*n* = 61, mean age = 19.05, SD = 1.86), transcript (*n* = 61, mean age = 18.70, SD = 1.23), and still image (*n* = 69, mean age = 19.19, SD = 2.75). Participants in each group were similar in their reported gender and cultural background (see Table 2). Two participants in the audio with video group and one participant in the transcript group self-reported a diagnosis of ASD. Therefore, they were excluded from the participant count and analyses. There were no other exclusion criteria for rating participants.

**Table 2:** Demographic information of rating participants.

	**Audio with video (*n* = 93)**	**Audio only (*n* = 62)**	**Video only (*n* = 61)**	**Transcript (*n* = 61)**	**Still image (*n* = 69)**	**Total sample (*n* = 346)**
**Variable**	***n*** **(%)**	***n*** **(%)**	***n*** **(%)**	***n*** **(%)**	***n*** **(%)**	***n*** **(%)**
**Gender**						
Men	20 (22%)	11 (18%)	16 (26%)	13 (21%)	11 (16%)	71 (21%)
Women	73 (78%)	51 (82%)	45 (74%)	48 (79%)	58 (84%)	275 (79%)
**Cultural background**						
Black/African American	3 (3%)	4 (7%)	2 (3%)	1 (2%)	4 (6%)	14 (4%)
East Asian	17 (18%)	11 (18%)	11 (18%)	10 (16%)	9 (13%)	58 (17%)
Indigenous	1 (1%)	2 (3%)	5 (8%)	3 (5%)	4 (6%)	15 (4%)
Latin American	2 (2%)	1 (2%)	3 (5%)	3 (5%)	1 (1%)	10 (3%)
South Asian	16 (17%)	8 (13%)	5 (8%)	7 (11%)	10 (14%)	46 (13%)
Southeast Asian	6 (7%)	10 (16%)	4 (7%)	7 (11%)	7 (10%)	34 (10%)
West Asian	6 (7%)	5 (8%)	5 (8%)	4 (7%)	8 (12%)	28 (8%)
White/European	42 (45%)	21 (34%)	26 (43%)	26 (43%)	26 (38%)	141 (41%)
**Autistic relatives**						
First-Degree	2 (2%)	1 (2%)	0	1 (2%)	0	4 (1%)
Second-Degree	1 (1%)	0	1 (2%)	3 (5%)	2 (3%)	7 (2%)
Third-Degree	6 (7%)	3 (5%)	4 (7%)	2 (3%)	4 (6%)	19 (6%)

First-degree relatives included parents and siblings; Second-degree relatives included grandparents, aunts and uncles, and nieces and nephews; Third-degree relatives included first cousins.

### 2.2. Materials

#### 2.2.1. Demographic information

The demographic information of participants was collected using a parent-report questionnaire for child stimulus participants and a self-report questionnaire for adult rating participants. Questions on both versions of the form inquired about the participant's sex, date of birth, cultural background, family or individual income, parental or individual education level, physical and mental health conditions, and family history of ASD diagnoses.

#### 2.2.2. Wechsler abbreviated scale of intelligence, 2nd edition (WASI-II)

The WASI-II (23) is a brief measure of cognitive ability for individuals aged between 6 and 90 years old. The Full-Scale IQ-2 (FSIQ-2), which measures verbal comprehension and perceptual reasoning, was administered to the child stimulus participants by graduate students in the Clinical Psychology program at Simon Fraser University. The reliability coefficient for the FSIQ-2 is good (0.93) for children aged between 6 and 16 years old. The WASI-II also has good interrater reliability for the Matrix Reasoning (0.99) and Vocabulary (0.95) subtests, which are used to calculate the FSIQ-2. The WASI-II was used in the current study to assess the equivalency of the intellectual ability of autistic and non-autistic child stimulus participants.

#### 2.2.3. Social responsiveness scale, 2nd edition (SRS-2)

The SRS-2 (24) is a standardised parent-report measure of autism symptom severity. The SRS-2 contains 65 items scored on a 4-point Likert scale (1 = Not True to 4 = Almost Always True) and produces two domain scores (Social Communication and Interaction; Restricted Interests and Repetitive Behaviour) and a total score. Raw domain and total scores are converted into *t*-scores, with *t*-scores ≥76 indicating a severe range of symptom severity, scores 66–75 indicating a moderate range of symptom severity, scores 60–65 indicating a mild range of symptom severity, and t-scores ≤ 59 indicating that the person is within typical limits. The SRS-2 demonstrated high internal consistency (Cronbach's α=0.95) in a standardisation study with 1,014 children. In the current study, SRS-2 was administered to corroborate the diagnoses of the autistic stimulus participants and assess for group differences in autism symptom severity. The reliability of the SRS-2 total score was good (Cronbach's α = 0.977, McDonald's ω = 0.979) within the current sample.

#### 2.2.4. Autism spectrum quotient (AQ)

The AQ is a self-report and parent-report measure of autistic traits (25). Fifty statements are answered on a 4-point Likert scale (1 = Definitely Agree to 4 = Definitely Disagree) and loaded onto five factors associated with autism: attention switching, attention to detail, social skills, communication, and imagination. The AQ self-report was administered to rating participants, and the parents of children aged 6–11 years old were asked to fill out the AQ-Child report (26). The sum of all questions yields the AQ total score, which provides an overall score of autistic traits for the individual, where higher scores represent a higher level of autistic traits. A cutoff score of 32 correctly identifies 80% of autistic adults (25) and 86% of autistic children (27). In the current study, the Autism Spectrum Quotient: Children's Version (AQ-Child) was utilised to confirm the diagnoses of participants with autism spectrum disorder and to compare the autistic traits between groups. The AQ self-report was administered to adults to evaluate the possible relationship between autistic traits and bias against autism. Four adult rating participants in the current study were found to have an AQ total score ≥32 (one in the audio with video group, one in the video only group, one in the transcript group, and one in the still image group). They were included in the analyses because they did not self-report a diagnosis of ASD. In the current study, the reliability of the AQ total score was acceptable for the adult rating participants (Cronbach's α = 0.714, McDonald's ω = 0.721) and child stimulus participants (Cronbach's α = 0.887, McDonald's ω = 0.900).

#### 2.2.5. Multidimensional social competence scale (MSCS)

The MSCS is a 77-item questionnaire that assesses social competence across the following seven domains: social motivation, social inferencing, demonstrating empathic concern, social knowledge, verbal conversation skills, nonverbal sending skills, and emotion regulation (28). The MSCS is scored on a 5-point Likert scale (1 = Not True or Almost Never True to 5 = Very True or Almost Always True), where higher scores represent greater levels of social competence. The MSCS was previously found to have good internal consistency (Cronbach's α = 0.795) among a sample of young adults. The psychometrically validated self-report MSCS (29) was completed by rating participants in the current study to evaluate the possible relationship between the social competence of the rater and bias against autism. The reliability of the MSCS total score for the current study was good (Cronbach's α = 0.926, McDonald's ω = 0.931).

#### 2.2.6. Quantity of contact

The quantity of previous contact with autistic people was assessed using a questionnaire originally developed by Holmes et al. (30) and adapted by Gardiner and Iarocci (31). Respondents answered yes or no to a series of 12 items that state varying degrees of closeness to an autistic person (e.g., “I have watched a movie or television show in which a character depicted a person with autism”; “I live with a person who has autism”; “I have autism”). The total number of “yes” responses served as the total quantity of contact score, ranging between 0 (no exposure) and 12 (exposure in many contexts). Following the procedures of Scheerer et al. (15) in the current study, participants who reported no real-world contact with an autistic person (i.e., “I have watched a movie or television show in which a character depicted a person with autism”, “I have never observed a person that I was aware had autism”, and “I have watched a documentary about autism”) were classified as having no direct contact with autistic people. The quantity of contact measure was used in the current study to evaluate the possible relationship between the amount of past contact with autistic people and bias against autism.

#### 2.2.7. Quality of contact

The quality of previous contact with autistic people was assessed using a questionnaire originally developed by McManus et al. (32) and adapted by Gardiner and Iarocci (31). Respondents answered six items using a 9-point Likert scale (1 = Strongly Disagree to 9 = Strongly Agree). This scale included items such as “Overall, I have had positive experiences with people with autism” and “The experiences I have had with people with autism have been fun”. The sum of all six items yielded a total score ranging between 6 and 54. Following the procedures of Scheerer et al. (15) only participants who indicated some direct contact with autistic people on the quantity of contact questionnaire received a quality of contact score. The quality of contact measure was used in the current study to evaluate the relationship between the quality of past experiences with autistic people and bias against autism. The reliability of the quality of contact total score for the current study was good (Cronbach's α = 0.898, McDonald's ω = 0.915).

#### 2.2.8. Social distance scale

The SDS is a self-reported measure of stigma towards autistic adults (33). Respondents answered six items on a 4-point Likert scale (1 = Definitely willing to 4 = Definitely unwilling), yielding a total score between 6 and 24. Higher total scores represent more autism stigma and greater social distance from autistic people. The SDS was used in the current study to evaluate the relationship between explicit autism stigma measured by this questionnaire and the autism bias score produced by the First Impressions Scale. The reliability of the SDS total score for the current study was good (Cronbach's α = 0.876, McDonald's ω= 0.881).

#### 2.2.9. First impression scale

A modified version of the FIS created by Sasson et al. (14) was presented against each stimulus participant and completed by each rating participant as a measure of explicit biases about autistic children. Similar to the original FIS, the modified FIS in the current study contained a series of 10 statements rated on a 4-point Likert scale (0 = Strongly disagree to 3 = Strongly agree). Six statements related to the stimulus child's characteristics (i.e., awkward, confident, trustworthy, aggressive/dominant, likeable, and smart) and four statements related to behavioural intentions towards the stimulus participant (i.e., willingness to live next to the child, the likelihood of hanging out with the child if they were the same age, comfort level sitting next to the child, and comfort level having a conversation with the child). Statements were re-phrased from the original FIS to accommodate the discrepancy in age between the rater and the stimulus participant (e.g., changing “This person is probably as smart as I am” to “This child is probably smart” and changing “I would hang out with this person in my free time” to “I would hang out with this child if I was their age”). Higher scores are indicative of a more positive impression of the stimulus participant; therefore, “awkward” and “aggressive/dominant” items were reverse scored as these characteristics are associated with more negative first impressions. Consistent with previous research (11), an “autism bias score” was calculated by taking each participant's mean rating of each FIS item for autistic stimulus participants and subtracting it from that of the non-autistic stimulus participants; positive values indicate a bias against autistic stimulus participants (i.e., higher ratings of non-autistic stimulus participants).

In the current study, the reliability of the FIS was good, as assessed by Cronbach's α and McDonald's ω: audio with video format (α = 0.986, ω = 0.988), audio only format (α = 0.990, ω = 0.990), video only format (α = 0.984, ω = 0.985), transcript format (α = 0.981, ω = 0.984), and still image format (α = 0.982, ω = 0.983).

### 2.3. Procedures

#### 2.3.1. Stimulus participants

The responses from stimulus participants that were used in the current study were elicited from one of two questions: “Tell me about your most favourite thing in the whole world.” and “Is there anything else you want to tell me about [favourite thing]?” During the interviews, participants were asked to sit in a chair across a table and 3 ft to the right of the researcher. A camera was concealed behind the researcher in a box and out of view of the participant. A Philips Voice Tracer DVT1150 audio recorder was placed between the participant and the interviewer to record the audio. During the interviews, participants discussed their favourite interests and behaviours associated with the interest and told the interviewer a storey about their interests. Following the interview, participants completed the WASI-II, and parents completed a demographic questionnaire, the AQ, and the SRS-2. Children were compensated with a t-shirt and toy/object ($5 value), and caregivers were compensated with entry into a draw for a $100 gift card.

For the audio with video, audio only, and video only formats, recordings lasted between 8 and 14 s for each group, similar to the range of 9–14 s in the stimuli created by Sasson et al. (14). The transcript format had the typed content of the child's utterance. Transcripts of each interview were typed verbatim by two research assistants and was checked a final time by the first author. Transcripts included filler words (e.g., “Umm”), self-corrections (e.g., “that happen- didn't happen”), informal pronunciations (e.g., “kinda”), and mispronunciations (e.g., “fright” instead of “fight”). The still image format included the first frame of the video where the child's head was upright, and their eyes were completely open.

#### 2.3.2. Rating participants

Participants reviewed a short description of the study (“You will be asked to make judgements about children observed through brief audio-visual formats as well as fill out questionnaires about your social behaviours and social motivation. The survey will take approximately 60 min to complete. You will be compensated with 2 credits for your participation”). If they chose to participate, they were shown the consent form on a Qualtrics web survey. After they completed the consent form, they were shown a separate Qualtrics web survey and randomly presented with one of the five audio-visual formats (audio with video, audio only, video only, transcript, and still image). Rating participants were not informed of the diagnostic status of participants. Each stimulus participant within that condition was presented one at a time in a random order. The FIS was completed by the rating participant for each stimulus participant. The rating participant then completed a demographics questionnaire, the AQ, the MSCS, the Quantity of Contact questionnaire, the Quality of Contact questionnaire (2), and the SDS. After completing the questionnaires, the participants were debriefed and compensated.

### 2.4. Analyses

Data were evaluated using SPSS Version 26. Descriptive statistics were reported for the main variables for child stimulus participants and adult rating participants.

To address our first hypothesis and assess whether the main variables (age, AQ total scores, MSCS total scores, quantity of contact score, quality of contact score, and SDS total scores) varied between ratings of audio-visual formats (audio with video, audio only, video only, transcript, and still image), one-way ANOVAs were conducted for each main variable. If the one-way ANOVA was statistically significant, Tukey's *post-hoc* analyses were conducted. If the assumption of homogeneity of variances was violated, Welch's ANOVA was used with Games-Howell *post-hoc* analyses. A Bonferroni correction was used for the one-way ANOVA analyses given the large number of comparisons (α = 0.0083). To assess for potential differences in the trait and behavioural ratings of autistic and non-autistic stimulus participants across different audio-visual formats, a 2 (autistic vs. non-autistic stimulus participant group) by 10 (rating for each FIS question) by 5 (audio-visual format) 3-way mixed-model ANOVA was conducted. A Greenhouse-Geisser correction was used when the assumption of sphericity was violated.

To address the second hypothesis, a correlation analysis was conducted between the main variables.

To address our exploratory hypothesis to better understand the differences between different audio-visual format ratings, we conducted *post-hoc* analyses using paired sample *t*-tests following the three-way mixed-model ANOVA. To control for multiple comparisons, a Bonferroni correction was used (α = 0.005).

## 3. Results

### 3.1. Descriptive statistics

Means and standard deviations of study variables were calculated for child stimulus participants and adult rating participants (see Tables 1, 3, respectively).

**Table 3:** Scores of rating participants.

	**Audio with video (*n* = 93)**	**Audio only (*n =* 62)**	**Video only (*n =* 61)**	**Transcript (*n =* 61)**	**Still image (*n =* 69)**	**Total sample (*n =* 346)**
**Variable**	**Mean (SD)**	**Mean (SD)**	**Mean (SD)**	**Mean (SD)**	**Mean (SD)**	**Mean (SD)**
Age	19.32 (1.69)	20.98 (4.98)	19.05 (1.86)	18.70 (1.23)	19.19 (2.75)	19.44 (2.84)
AQ total score	18.17 (4.87)	19.89 (4.38)	17.79 (5.27)	17.67 (5.05)	17.35 (5.66)	18.16 (5.10)
MSCS total score	295.16 (25.19)	285.18 (26.76)	295.26 (28.59)	293.79 (25.64)	3.6.03 (24.61)	295.32 (26.72)
Quantity of contact score	3.60 (2.15)	3.58 (1.82)	4.52 (2.17)	4.11 (2.17)	4.33 (2.05)	4.00 (2.10)
Quality of contact score	35.75 (9.95)	34.34 (8.70)	37.03 (10.65)	34.13 (10.20)	31.54 (11.83)	34.60 (10.42)
SDS total score	10.23 (3.72)	11.37 (4.08)	11.82 (4.06)	11.62 (4.10)	12.10 (4.77)	11.33 (4.17)

AQ, Autism-Spectrum Quotient; MSCS, Multidimensional Social Competence Scale; SDS, Social Distance Scale.

### 3.2. One-way ANOVA

A series of one-way ANOVAs were conducted to determine if age, AQ total scores (autistic traits), MSCS total scores (social competence), quantity of contact, quality of contact, and SDS total scores (explicit stigma) were different for raters across the five audio-visual formats. For the variable of age, homogeneity of variances was violated, as assessed by Levene's test for equality of variances (*p* = 0.003); therefore, a Welch's ANOVA was conducted. The one-way Welch ANOVA for participant age was significant [Welch's *F*_(4,158.876)_ = 4.136, *p* = 0.003]. A Games-Howell *post-hoc* analysis found that the audio only group was significantly older than the transcript group (Mean difference = 2.28, 95% CI [0.45, 4.10], *p* = 0.007). Other *post-hoc* comparisons did not survive correction for multiple comparisons (*p* > 0.0083).

For the variable of MSCS total scores, the homogeneity of variances was violated (*p* ≤ 0.001). Welch's ANOVA was significant [Welch's *F*_(4,162.629)_ = 5.467, *p* ≤ 0.001]. A Games-Howell *post-hoc* analysis found that the still image group had significantly higher total MSCS scores than the audio only group (mean difference = 20.85, 95% CI [8.37, 33.33], *p* ≤ 0.001). Other *post-hoc* comparisons did not survive correction for multiple comparisons (*p* > 0.0083).

The assumption of homogeneity of variances was met for the one-way ANOVAs for autistic traits, quantity of contact, quality of contact, and SDS total scores. The one-way ANOVAs for total AQ scores, quantity of contact, quality of contact, and total SDS scores were not significant (*p* > 0.0083).

### 3.3. Three-way mixed-model ANOVA

A 2 (autistic vs. non-autistic stimulus participant group) by 10 (rating) by 5 (audio-visual format) mixed-model ANOVA was conducted (see Table 4). There was homogeneity of variances, as assessed by Levene's test for equality of variances (*p* > 0.05). Mauchly's test of sphericity indicated that the assumption of sphericity was violated [$\chi_{\left(44\right)}^{2}$ = 661.588, *p* ≤ 0.001]; therefore, a Greenhouse-Geisser correction was reported. There was a statistically significant three-way interaction between the stimulus group, audio-visual format, and rating [*F*_(23.335, 1, 989.287)_ = 4.470, *p* ≤ 0.001, $\eta_{P}^{2}$ = 0.050]. The statistical significance of a simple two-way interaction was accepted at a Bonferroni-adjusted alpha level of 0.025. There was a significant two-way interaction between the stimulus group and audio-visual formats [*F*_(4,341)_ = 9.375, *p* ≤ 0.001, $\eta_{P}^{2}$ = 0.099] and between the stimulus group and rating [*F*_(5.834, 1, 989.287)_ = 16.067, *p* ≤ 0.001, $\eta_{P}^{2}$ = 0.045). There was a simple main effect of the stimulus group [*F*_(1,341)_ = 77.774, *p* ≤ 0.001, $\eta_{P}^{2}\;=$ 0.186], rating [*F*_(3.544, 1208.401)_ = 321.079, *p* ≤ 0.001, $\eta_{P}^{2}$ = 0.485], and audio-visual formats [*F*_(1,341)_ = 4.267, *p* = 0.002, $\eta_{P}^{2}$ = 0.048].

**Table 4:** Three-way mixed-model ANOVA.

	**Sum of squares**	***df***	**Mean square**	***F***	***p***	***Partial η^2^***
**Between-subject effects**						
Condition	28.3	4	7.076	4.267	0.002	0.048
Error (Condition)	565.5	341	1.658			
**Within-subject effects**						
Stimulus group	5.585	1	5.585	77.77	≤ 0.001	0.186
Error (Stimulus group)	24.49	341	0.072			
Rating	573.3	3.544	161.8	321.1	≤ 0.001	0.485
Error (Rating)	609.1	1,208	0.504			
Stimulus group x Rating	2.578	5.834	0.442	16.07	≤ 0.001	0.045
Stimulus group x Condition	2.693	4	0.673	9.375	≤ 0.001	0.099
Rating x Condition	20.04	14.18	1.414	2.804	≤ 0.001	0.032
Stimulus group x Rating x Condition	2.868	23.34	0.123	4.47	≤ 0.001	0.05

n = 346.

### 3.4. Pearson correlation

Pearson's correlations were used to investigate whether the characteristics of raters across all audio-visual formats were associated with autism bias scores (see Table 5). Total AQ scores were negatively correlated with autism bias scores [*r*_(344)_ = −0.276, *p* ≤ 0.001], indicating that raters with more autistic traits had a lower autism bias score. Similarly, quality of contact was negatively correlated with autism bias scores [*r*_(344)_ = −0.221, *p* ≤ 0.001], indicating that raters who reported more positive past experiences with autistic people also had a lower autism bias score.

**Table 5:** Pearson correlations.

	**1**	**2**	**3**	**4**	**5**	**6**	**7**
1. Autism bias score	-						
2. Age	−0.008	-					
3. AQ total score	−0.276^**^	0.074	–				
4. MSCS total score	0.311^**^	−0.036	−0.447^**^	–			
5. Quantity of contact	0.094	−0.003	−0.040	0.050	-		
6. Quality of contact	−0.221^**^	−0.046	0.015	−0.160^*^	0.325^**^	-	
7. SDS score	0.340^**^	0.033	0.009	0.012	0.096	−0.081	-

n = 346. ^*^p <0.05, ^**^p ≤ 0.001.

MSCS, Multidimensional Social Competence Scale; AQ, Autism-Spectrum Quotient; SDS, Social Distance Scale.

On the other hand, the total MSCS scores were positively correlated with autism bias scores [*r*_(344)_ = 0.311, *p* ≤ 0.001), indicating that raters with higher social competence had higher autism bias scores. Similarly, total SDS scores were also positively correlated with autism bias scores [*r*_(344)_ = 0.340, *p* ≤ 0.001], indicating that raters with higher explicit autism stigma had higher autism bias scores. Neither age nor quantity of contact were significantly correlated to autism bias scores.

### 3.5. *Post-hoc* paired sample *t*-tests

Follow-up paired-sample *t*-tests were conducted to elucidate the interaction effects. Based on the audio-visual format interaction, the stimulus group rated autistic children less favourably than non-autistic children in the audio with video [*t*_(92)_ = −7.750, *p* ≤ 0.001, *d* = 0.12], audio only [*t*_(61)_ = −5.909, *p* ≤ 0.001, *d* = 0.13], video only [*t*_(60)_ = −3.213, *p* ≤ 0.001, *d* = 0.12], and transcript [*t*_(60)_ = −4.173, *p* ≤ 0.001, *d* = 0.10] formats, but not the still image format [*t*_(68)_ = 0.584, *p* = 0.281, *d* = 0.13].

**Figure 1:**
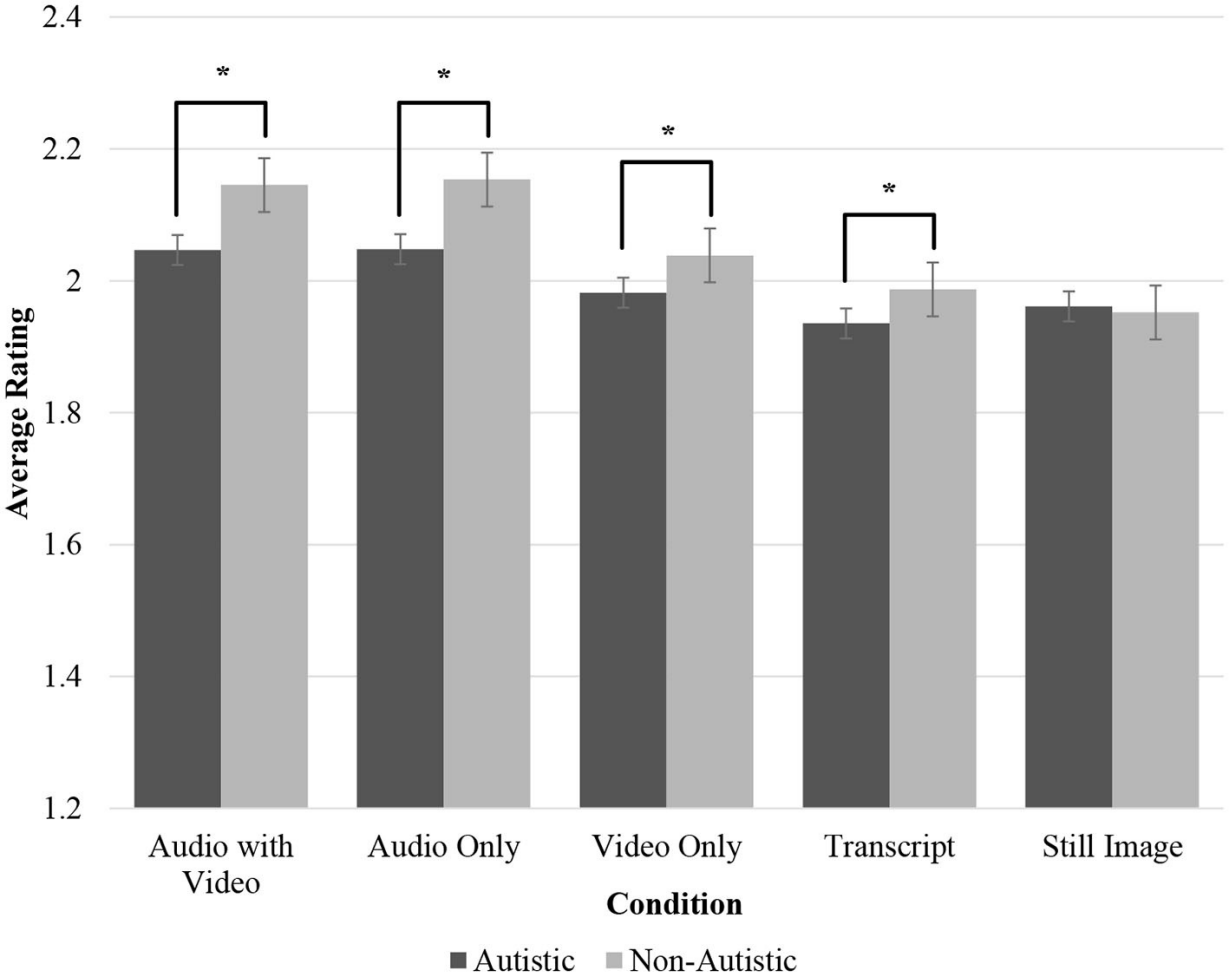
Average ratings of stimulus participants across conditions by group. The significance of the *post-hoc* paired-sample *t*-tests of the stimulus group by audio-visual format at *p* ≤ 0.001 is indicated by an asterisk (^*^). Error bars represent standard errors.

Similarly, autistic children were rated significantly less favourably than non-autistic children on ratings of awkwardness [*t*_(345)_ = −4.315, *p* ≤ 0.001, *d* = 0.27], trustworthiness [t_(345)_ = −5.027, *p* ≤ 0.001, *d* = 0.20], aggression [t_(345)_ = −8.974, *p* ≤ 0.001, *d* = 0.21], likability [t_(345)_ = −6.595, *p* ≤ 0.001, *d* = 0.20], willingness to live near the child [t_(345)_ = −5.834, *p* ≤ 0.001, *d* = 0.19), willingness to hang out with child if they were the same age [t_(345)_ = −8.856, *p* ≤ 0.001, *d* = 0.26], comfort level sitting next to the child [t(345) = −5.357, *p* ≤ 0.001, *d* = 0.19], and comfort level conversing with the child [t*(345)* = −6.946, *p* ≤ 0.001, *d* = 0.19] but not for ratings of confidence (*p* = 0.009) or intelligence (*p* = 0.008) given the Bonferroni correction (see Figure 1).

The three-way interaction between stimulus group, rating, and the audio-visual format identified that autistic children were rated significantly less favourably in the audio with video format on awkwardness (*p* ≤ 0.001), trustworthiness (*p* ≤ 0.001), aggressiveness (*p* ≤ 0.001), likeability (*p* ≤ 0.001), and willingness to live next to (*p* ≤ 0.001), hang out with the child if they were the same age (*p* ≤ 0.001), sit next to the child (*p* ≤ 0.001), and have a conversation with the child (*p* ≤ 0.001) (see Supplementary Table S1). In the audio only category, autistic children were rated significantly less favourably on confidence (*p* ≤ 0.001), trustworthiness (*p* ≤ 0.001), aggressiveness (*p* ≤ 0.001), likeability (*p* ≤ 0.001), and willingness to live next to (*p* ≤ 0.001), hang out (*p* ≤ 0.001), sit next to (*p* ≤ 0.001), and have a conversation (*p* ≤ 0.001). In the video-only category, autistic children were rated less favourably than non-autistic children on aggressiveness (*p* ≤ 0.001), likeability (*p* ≤ 0.001), and willingness to sit next to (*p* ≤ 0.001) and have a conversation (*p* ≤ 0.001). In the transcript condition, autistic children were rated significantly less favourably than non-autistic children on awkwardness (*p* ≤ 0.001), trustworthiness (*p* ≤ 0.001), likeability (*p* ≤ 0.001), and willingness to hang out (*p* ≤ 0.001). There were no significant differences in ratings of autistic and non-autistic children within the still image format.

## 4. Discussion

Previous research on first impressions of autistic children and adults indicated that autistic people are rated less favourably than non-autistic people following brief exposure to de-contextualised “thin slices” of social behaviour. The current study investigated the first impressions of autistic and non-autistic children across different audio-visual formats (i.e., audio with video, audio only, video only, transcript, and still image) as judged by non-autistic adults. The relationships between first impression ratings and the rater's own characteristics (i.e., autistic traits, social competence, quality and quantity of past experiences with autistic people, and explicit autism stigma) were also investigated.

Consistent with the first hypothesis, autistic children were rated less favourably than non-autistic children in terms of traits of the child and the rater's behavioural intentions towards the child. However, these first impression ratings of autistic and non-autistic children differed between audio-visual formats. Raters viewing the stimulus participants through audio with video and audio only formats rated autistic children less favourably on most traits and all behavioural intentions towards the child. In the category without audio information (i.e., video only format), there were fewer differences in ratings between autistic and non-autistic children and even fewer differences when raters evaluated the transcript of the conversation. No significant differences were found in the ratings of still images between autistic and non-autistic children.

The current findings partially replicate those by Sasson et al. (14) in ratings of autistic and non-autistic adults. In their study, non-autistic adults rated autistic adults more negatively in all formats of audio and/or visual information, including the still image format. However, Sasson et al. found no differences in ratings in the transcript format. They posit that these differences may have occurred due to the potential atypicalities in physical presentation, non-verbal communication, and paralinguistic features of speech, such as inflexion. Compared to non-autistic people, autistic people are more likely to have unusual prosody in their speech (34). In social interactions, the modulation of prosody is related to social competency, as normative social interactions often include the identification and transmission of nonverbal information such as emotions, attitudes, and intentions (35, 36). Raters in the present study might have identified the prosodic peculiarities of autistic stimulus participants when audio information was available, contributing to their more negative ratings of autistic children. However, paralinguistic features of speech are just one of several factors contributing to social competency, indicating that atypical prosody may be one of several mechanisms by which negative impressions are formed about autism.

Autistic children in audio-visual formats containing visual information, except for the still image format, were rated more negatively than non-autistic children in the current study. The physical movements and non-verbal communication patterns of autistic children were often observably different from those of non-autistic children, with characteristics such as reduced eye contact and gesture use (37). These differences may be perceived as peculiar by the rater and, thus, were rated more negatively than those with more typical non-verbal communication. A still image of a child may not provide enough information about that child's non-verbal communication for a negative first impression to be formed on that basis. However, Sasson et al. (14) found that autistic adults were rated more negatively than non-autistic adults in their still image condition. A similar rating between autistic and non-autistic children in the present study may be due to how the still images were created. Given that the still image was created using the first frame of the video where the child's eyes were open and their head was upright, aberrations of social communicative norms (e.g., looking down or away during a conversation) may have been missed. Future studies would benefit from using a random sampling of still images from across the video stimuli (instead of the first frame) to account for this potential limitation.

An interesting finding in the current study was the difference in ratings between autistic and non-autistic children in the transcript format. Autistic children were rated more negatively on awkwardness, trustworthiness, likeability, and willingness to spend time together. Given that the first impression stimuli were created from the interview of children talking about their interests, these findings might represent negative first impressions of how autistic children's interests were expressed, as the interests of autistic and non-autistic stimulus participants were similar in the current study. While an analysis of the content of speech is beyond the scope of the current study, autistic children have been found to use different “fillers” in language compared to non-autistic children, such as using “um” less frequently (38, 39). Autistic children may also have difficulties articulating a spoken narrative coherently and cohesively, with less complex language and greater repetitions in their speech than non-autistic children (40). As the raters in the current study viewed the verbatim transcriptions of the stimulus participants' speech, it is possible that these qualities of autistic children's speech were viewed as more awkward and negative than non-autistic children. Future research is needed to investigate negative attitudes towards autism and stigma based on the possible peculiarities in the content of speech and the discourse markers of autistic children.

Consistent with the second hypothesis, the rater's explicit autism stigma in the current study was found to be related to higher autism bias on the first impression task. These findings are similar to those of Morrison et al. (16). Using only the autistic adult stimuli by Sasson et al. (14), Morrison et al. (16) examined the characteristics of non-autistic college students on first impressions. They found that higher autism stigma measured by the SDS predicted less favourable ratings of autistic adults on most traits. Research by Aubé et al. (41) examining elementary school-age children's attitudes towards autistic children found high levels of explicit autism stigma in younger children but less explicit negative attitudes in older children. However, children across different elementary school ages held similar levels of implicit negative attitudes towards autism, measured by a faster spontaneous decision to avoid autistic children and approach non-autistic children when presented with videos featuring both autistic and non-autistic children. Implicit attitudes are evaluations arising from experiences which may occur outside of conscious awareness (42). Aubé et al.'s (41) findings indicate that, even if explicit attitudes improve, implicit attitudes may still remain. Educating people about autism can lead to improvements in explicit attitudes towards autism (15, 43), but implicit attitudes remain a better real-world predictor of non-deliberate and impulsive behaviour than explicit attitudes (44, 45). Unfortunately, implicit attitudes may be less susceptible to change by improving autism knowledge (46), which may explain why autistic people experience discrimination and victimisation despite greater societal autism knowledge and improving explicit attitudes towards autism (47, 48). Therefore, it is of interest for future research to examine the nature of implicit attitudes towards autism and explore the mechanisms by which implicit attitudes may be improved.

Consistent with the second hypothesis, higher social competence scores of the raters were associated with greater bias towards autistic children. Furthermore, higher levels of the raters' autistic traits were related to lower bias towards autistic children. Finally, across all raters, more negative ratings of autistic children were associated with higher levels of the rater's social competence and explicit stigma. In contrast, more positive ratings of autistic children were associated with higher levels of the rater's autistic traits and quality of past experiences with autistic people.

Individuals who self-reported greater social competence may be more perceptive to behaviours and parts of interactions that are divergent from the norm, such as peculiarities in body language and gaze, thereby leading to more negative evaluations. In contrast, individuals with greater autistic traits may be comparatively less perceptive to these social divergences. In addition, individuals with greater autistic traits may have a better implicit understanding of the experiences of children who also show such traits, potentially constructed or bolstered through personal experience. Therefore, they may be more empathetic, sympathetic, or less judgemental when perceiving others with similar experiences. Finally, there may be other unmeasured characteristics of individuals with autistic traits that positively influence their openness to differences of others more broadly.

It is worth noting, however, that a recent systematic review and meta-analysis on the characteristics of non-autistic adults on autism stigma by Kim et al. (20) found no significant correlation between the autistic traits of the rater and their attitudes towards autistic people. In the current study, higher autistic traits of the rater measured by their AQ were related to lower autism stigma measured by the autism bias score derived from the FIS; but AQ scores were not related to a measure of explicit autism stigma determined by the SDS. It may be that FIS is closer to an implicit measure of autism stigma, as the questions are less overt when querying for autism stigma than the SDS, and rating participants were unaware of the diagnostic status of stimulus participants. Previous research shows a relationship between AQ scores and implicit bias, where participants with higher autistic traits demonstrated less implicit bias towards autism (47).

Past positive experiences of raters with autism were related to lower bias towards autistic children in the current study, although the number of past experiences with autistic people was not. Negative evaluations of autistic people may be mitigated or improved by having more positive past experiences and by improving acceptance and openness towards autistic people (31). Simply having more experiences with autistic people may be insufficient to change attitudes towards autism if individuals still lack knowledge about autism or hold stereotyped or prejudiced views (49). Indeed, improving autism knowledge is related to lower bias towards autistic adults (15) and autistic children (50), stressing the importance of providing accurate autism knowledge in addressing autism stigma.

The overall finding that non-autistic adults hold more negative perceptions of autistic children than non-autistic children has implications for the acceptance and de-stigmatisation of autism within society. In the life of an autistic child, adults play a crucial role in nurturing and supervising many of the contexts in which the child interacts, such as the classroom and community spaces. Successful inclusion and acceptance of autistic children in these spaces depends on the de-stigmatisation of autism and the acceptance and humanisation of neurodiverse people. Autistic individuals in several studies have reported animosity, prejudice, and stigma against autism in general and against themselves as autistic people (11, 51, 52), which can have deleterious effects on mental health, self-perception, and wellbeing (53). For autistic children who are already vulnerable to social and mental health difficulties, enduring negative attitudes towards autism held by adults has the potential to undercut their dignity, growth, and participation in educational and social environments. Furthermore, attitudes held by adults can influence the formation of similar attitudes in children (54, 55), which may influence how children interact with disabled or otherwise different peers (56). Awareness of one's attitudes towards autism is an important step in addressing the transmission of negative attitudes to children and the possible consequences of the inclusion or exclusion of autistic children.

### 4.1. Limitations

A few limitations of the study are worth noting. First, it is unclear how these results translate to real-world interactions with autistic children. Although participants reported increased explicit autism stigma and a negative bias towards autism, other factors, such as the situational context and their relationship to the child, may more strongly affect the way an adult interacts with an autistic child.

Second, autism knowledge was not measured. The quality and quantity of past autism contact cannot be assumed to be equivalent having knowledge about autism. It is possible that certain raters in the current study were more knowledgeable of how autism presents in children, and thus, this knowledge may have influenced their first impression judgements. In previous studies, mixed results were found whereby one study showed an association between more positive attitudes towards autism and greater autism knowledge, (19) while another found no such association (50).

Third, the AQ self-report measure of autistic traits in the current study was normed on a predominantly male sample. Given that the current study's sample was predominantly female, the autistic traits of these participants may not have been sufficiently assessed. The development of more comprehensive questionnaires and diverse validation of existing measures may be of value for future research.

Fourth, the stimulus participants did not have intellectual disabilities, and they all used verbal language as a primary means of communication. Within the population of autistic children, approximately 38% have a co-occurring diagnosis of intellectual disability (57), and approximately 30% are non-speaking (58, 59). Therefore, the results of the current study are limited in their generalisability insofar as it only includes a particular presentation of ASD and does not include the wider range of abilities present in the diverse population of autistic children. It is possible that the stigma against non-speaking autistic children is greater than the stigma against speaking autistic children, given the importance verbal language has in social interactions. Alternatively, raters might more easily recognise a non-speaking child as autistic, which may potentially soften their first impression judgements. Future research should strive to include a more diverse and representative sample of autistic children with varying language and intellectual abilities to best understand autism stigma, as it affects all autistic children.

Finally, there is a possibility of selection bias in the current study's first impression stimuli compared to the greater sample of interviews by Boucher (21). The inclusion criteria for the stimulus participants required a specific set of criteria to protect the identity of the child and to ensure consistency between visual and audio formatting. The factors that excluded certain interviews (e.g., exiting one's seat and stepping out of frame when not appropriate to do so and speaking in single-word or brief phrases interrupting the recording of an 8 s clip) may have had an impact on first impression ratings. The relations between these behaviours and communicative norm violations in relation to the formation of first impressions and autism stigma could be examined in the future.

### 4.2. Conclusion

The study successfully isolated the effect of audio-visual formats (i.e., auditory, visual, and content cues within a de-contextualised conversational segment) and examined the effect of a number of the non-autistic rater's personal characteristics (i.e., explicit stigma, social competence, autistic traits, and past experiences with autistic people) on the formation of first impressions of autistic and non-autistic children. Our findings were generally consistent with those of previous studies, and the proposed hypotheses were supported. Our findings suggest that visual and auditory cues may trigger negative first impressions of autistic children in non-autistic adults but that the personal characteristics of the observer also play a role.

This study supports the robust nature of the negative first impression bias towards autistic individuals. Identifying which factors are most influential and how they may be addressed in prevention and intervention programs are important next steps in research aimed at counteracting harmful perceptions and attitudes about autistic people.

## Data availability statement

The raw data supporting the conclusions of this article will be made available by the authors, without undue reservation.

## Ethics statement

The studies involving humans were approved by Simon Fraser University Department of Research Ethics. The studies were conducted in accordance with the local legislation and institutional requirements. Written informed consent for participation in this study was provided by the participants' legal guardians/next of kin.

## Author contributions

TB, NS, and GI designed the study. TB and NS created the novel stimuli and collected the study data. TB, JL, and NS conducted the analysis and interpretation of the data. TB wrote the first draft of the manuscript in consultation with JL, NS, and GI. All authors provided feedback on the manuscript and approved the final version of the manuscript.
