# Supplementary material for: Negative first impression judgements of autistic children by non-autistic adults
Source: Front Psychiatry. 2023 Oct 6;14:1241584. doi: 10.3389/fpsyt.2023.1241584 (PMC10587469; doi:10.3389/fpsyt.2023.1241584)
Supplement: Supplementary file 1 [file Data_Sheet_1.docx]

Supplementary Material

**Negative First Impression Judgements of Autistic Children by Non-Autistic Adults**

Troy Q. Boucher, Julia N. Lukacs, Nichole E. Scheerer, and Grace Iarocci*

*** Correspondence:** Grace Iarocci: addl@sfu.ca

| **Table S1** *Paired-Sample t-Tests of Ratings of Autistic and Non-Autistic Stimulus Participants* | | | | | | | | | | | | | | | |
| --- | --- | --- | --- | --- | --- | --- | --- | --- | --- | --- | --- | --- | --- | --- | --- |
|  | Audio & Video Condition (*n* = 93) | | | Audio Only Condition (*n* = 62) | | | Video Only Condition (*n* = 61) | | | Transcript Condition (*n* = 61) | | | Still Image Condition (*n* = 69) | | |
|  | *t* | *p* | *d* | *t* | *p* | *d* | *t* | *p* | *d* | *t* | *p* | *d* | *t* | *p* | *d* |
| Awkward | -4.60 | ≤ .001 | .29 | -1.92 | .030 | .30 | 0.06 | .477 | .29 | -2.90 | ≤ .001 | .23 | 0.18 | .431 | .23 |
| Confident | 1.43 | .078 | .30 | 3.68 | ≤ .001 | .29 | 1.13 | .131 | .25 | -0.35 | .363 | .22 | -1.32 | .096 | .22 |
| Trustworthy | -5.09 | ≤ .001 | .18 | -3.08 | ≤ .001 | .17 | -2.09 | .020 | .18 | -3.45 | ≤ .001 | .19 | 1.22 | .113 | .24 |
| Aggressive/ Dominant | -6.77 | ≤ .001 | .22 | -5.77 | ≤ .001 | .24 | -3.43 | ≤ .001 | .18 | -2.29 | .013 | .17 | -1.57 | .061 | .22 |
| Likeable | -6.00 | ≤ .001 | .20 | -4.61 | ≤ .001 | .22 | -3.31 | ≤ .001 | .18 | -3.03 | ≤ .001 | .20 | 2.20 | .016 | .19 |
| Smart | -2.57 | .006 | .21 | -1.78 | .040 | .21 | -1.63 | .054 | .20 | -0.68 | .249 | .20 | 1.72 | .045 | .19 |
| Live Next To | -4.49 | ≤ .001 | .17 | -4.30 | ≤ .001 | .22 | -2.72 | .004 | .18 | -1.00 | .160 | .14 | -0.43 | .336 | .21 |
| Hang Out | -7.89 | ≤ .001 | .25 | -6.59 | ≤ .001 | .28 | -2.59 | .006 | .23 | -3.99 | ≤ .001 | .22 | 1.43 | .079 | .21 |
| Sit Next To | -3.46 | ≤ .001 | .20 | -4.26 | ≤ .001 | .19 | -3.80 | ≤ .001 | .18 | -0.88 | .191 | .14 | 0.15 | .441 | .22 |
| Have a Conversation With | -4.82 | ≤ .001 | .17 | -5.57 | ≤ .001 | .21 | -3.79 | ≤ .001 | .18 | -2.61 | .006 | .16 | 0.29 | .387 | .22 |
| *Note*. *df* for each comparison is *n*-1 for each group. | | | | | | | | | | | | | | | |

| **Table S2** *Mean (Standard Deviation) for Item- and Summary-Level Scores on the First Impression Scale (FIS)* | | | | | | | | | | | | |
| --- | --- | --- | --- | --- | --- | --- | --- | --- | --- | --- | --- | --- |
|  | Audio with Video (*n* = 93) | | Audio Only (*n* = 62) | | Video Only (*n* = 61) | | Transcript (*n* = 61) | | Still Image (*n* = 69) | | Total Sample (*n* = 346) | |
|  | Autistic | Non-Autistic | Autistic | Non-Autistic | Autistic | Non-Autistic | Autistic | Non-Autistic | Autistic | Non-Autistic | Autistic | Non-Autistic |
| Awkward | 1.58 (0.87) | 1.72 (0.80) | 1.66 (0.83) | 1.73 (0.82) | 1.59 (0.83) | 1.59 (0.77) | 1.43 (0.77) | 1.51 (0.81) | 1.62 (0.82) | 1.62 (0.80) | 1.58 (0.83) | 1.64 (0.80) |
| Confident | 1.79 (0.77) | 1.75 (0.75) | 1.87 (0.79) | 1.74 (0.82) | 1.59 (0.84) | 1.55 (0.73) | 1.59 (0.76) | 1.60 (0.77) | 1.75 (0.81) | 1.79 (0.75) | 1.73 (0.80) | 1.69 (0.77) |
| Trustworthy | 1.96 (0.56) | 2.05 (0.55) | 1.96 (0.59) | 2.03 (0.57) | 1.79 (0.61) | 1.84 (0.58) | 1.91 (0.59) | 2.00 (0.56) | 1.78 (0.70) | 1.74 (0.69) | 1.89 (0.61) | 1.94 (0.61) |
| Aggressive/ Dominant | 2.34 (0.74) | 2.49 (0.67) | 2.29 (0.80) | 2.47 (0.68) | 2.25 (0.70) | 2.33 (0.63) | 2.31 (0.68) | 2.36 (0.67) | 2.03 (0.79) | 2.07 (0.74) | 2.25 (0.75) | 2.35 (0.69) |
| Likeable | 1.94 (0.58) | 2.06 (0.56) | 1.92 (0.65) | 2.05 (0.59) | 1.94 (0.57) | 2.02 (0.55) | 1.84 (0.56) | 1.92 (0.55) | 1.93 (0.62) | 1.89 (0.59) | 1.92 (0.60) | 1.99 (0.57) |
| Smart | 1.95 (0.65) | 2.01 (0.61) | 1.95 (0.70) | 2.00 (1.61) | 1.86 (0.58) | 1.90 (0.59) | 1.76 (0.71) | 1.78 (0.67) | 1.87 (0.63) | 1.83 (0.62) | 1.88 (0.66) | 1.91 (0.63) |
| Live Next To | 2.34 (0.71) | 2.42 (0.67) | 2.31 (0.74) | 2.42 (0.65) | 2.33 (0.66) | 2.39 (0.64) | 2.27 (0.65) | 2.29 (0.65) | 2.30 (0.73) | 2.31 (0.70) | 2.31 (0.70) | 2.37 (0.66) |
| Hang Out | 1.54 (0.75) | 1.75 (0.75) | 1.63 (0.79) | 1.86 (0.73) | 1.61 (0.71) | 1.68 (0.70) | 1.51 (0.75) | 1.63 (0.72) | 1.47 (0.81) | 1.43 (0.78) | 1.55 (0.76) | 1.67 (0.75) |
| Sit Next To | 2.40 (0.69) | 2.48 (0.65) | 2.37 (0.72) | 2.47 (0.63) | 2.29 (0.68) | 2.38 (0.64) | 2.27 (0.66) | 2.29 (0.67) | 2.32 (0.77) | 2.31 (0.77) | 2.34 (0.71) | 2.39 (0.68) |
| Have a Conversation | 2.37 (0.72) | 2.46 (0.66) | 2.31 (0.76) | 2.46 (0.65) | 2.28 (0.69) | 2.37 (0.64) | 2.20 (0.69) | 2.25 (0.68) | 2.28 (0.80) | 2.27 (0.79) | 2.30 (0.74) | 2.37 (0.69) |

| **Table S2, Continued** | | | | | | | | | | | | |
| --- | --- | --- | --- | --- | --- | --- | --- | --- | --- | --- | --- | --- |
|  | Audio with Video (*n* = 93) | | Audio Only (*n* = 62) | | Video Only (*n* = 61) | | Transcript (*n* = 61) | | Still Image (*n* = 69) | | Total Sample (*n* = 346) | |
|  | Autistic | Non-Autistic | Autistic | Non-Autistic | Autistic | Non-Autistic | Autistic | Non-Autistic | Autistic | Non-Autistic | Autistic | Non-Autistic |
| Trait Score | 1.93 (0.74) | 2.01 (0.71) | 1.94 (0.75) | 2.00 (0.73) | 1.84 (0.73) | 1.87 (0.70) | 1.81 (0.74) | 1.86 (0.73) | 1.83 (0.74) | 1.82 (0.72) | 1.87 (0.74) | 1.92 (0.72) |
| Behavioural Intention Score | 2.17 (0.80) | 2.28 (0.75) | 2.15 (0.81) | 2.30 (0.71) | 2.13 (0.75) | 2.21 (0.72) | 2.06 (0.76) | 2.11 (0.73) | 2.09 (0.86) | 2.08 (0.85) | 2.12 (0.80) | 2.20 (0.76) |
| FIS Total Score | 2.02 (0.77) | 2.12 (0.74) | 2.03 (0.79) | 2.12 (0.74) | 1.95 (0.75) | 2.00 (0.73) | 1.91 (0.76) | 1.96 (0.74) | 1.94 (0.80) | 1.93 (0.78) | 1.97 (0.78) | 2.03 (0.75) |
| *Note*. The mean is reported atop the standard deviation, which is in brackets. "Trait Score" is derived from the following scores: Awkward, Confident, Trustworthy, Aggressive/Dominant, Likeable, and Smart. "Behavioural Intention Score" is derived from the following scores: Live Next To, Hang Out, Sit Next To, and Have a Conversation. The “FIS Total Score” is derived from scores on all ten items of the FIS. | | | | | | | | | | | | |
